# Population-Based Approaches to Mental Health: History, Strategies, and Evidence

**DOI:** 10.1146/annurev-publhealth-040119-094247

**Published:** 2020-01-06

**Authors:** Jonathan Purtle, Katherine L. Nelson, Nathaniel Z. Counts, Michael Yudell

**Affiliations:** 1 Department of Health Management and Policy, Dornsife School of Public Health, Drexel University, Philadelphia, Pennsylvania 19104, USA;; 2 Mental Health America, Alexandria, Virginia 22314, USA; 3 Department of Community Health and Prevention, Dornsife School of Public Health, Drexel University, Philadelphia, Pennsylvania 19104, USA

**Keywords:** mental health, population health, public policy, public health practice, health care system design, psychiatric epidemiology

## Abstract

There is growing recognition in the fields of public health and mental health services research that the provision of clinical services to individuals is not a viable approach to meeting the mental health needs of a population. Despite enthusiasm for the notion of population-based approaches to mental health, concrete guidance about what such approaches entail is lacking, and evidence of their effectiveness has not been integrated. Drawing from research and scholarship across multiple disciplines, this review provides a concrete definition of population-based approaches to mental health, situates these approaches within their historical context in the United States, and summarizes the nature of these approaches and their evidence. These approaches span three domains: (*a*) social, economic, and environmental policy interventions that can be implemented by legislators and public agency directors, (*b*) public health practice interventions that can be implemented by public health department officials, and (*c*) health care system interventions that can be implemented by hospital and health care system leaders.

## INTRODUCTION

Approaches to addressing mental health issues in society have overwhelmingly focused on the provision of clinical services to individuals, not on fostering conditions that promote positive mental health, mental health promotion, or the primary prevention of mental illness. Although clinical mental health services dramatically improve the lives of many people and their families (13), there are several reasons why the provision of clinical services in isolation is a suboptimal approach to maximizing the mental health of a population.

**Positive mental health:**

sense of well-being, capacity to enjoy life, and the ability to deal with challenges—not only the absence of a mental health illness or condition

**Mental health promotion:**

actions to improve quality of life and well-being, as opposed to ameliorating mental illness symptoms and mental health deficits

**Primary prevention of mental illness:**

actions that address factors that might cause mental illnesses to avert their development

Most people who need mental health services do not receive them (137), in part because of workforce shortages. Estimates indicate that an additional 15,400 psychiatrists and 57,490 psychologists are needed by 2025 to meet the mental health service demand of the US population (74). Among people who do receive mental health services, there is a moderate likelihood that the services provided will not be evidence-based, implemented with fidelity, or effective (13, 26, 100). For these reasons, population-level increases in mental health service utilization are not associated with improvements in population mental health status (79). Finally, evidence suggests that much of the population burden of mental health problems can be prevented by reducing exposure to traumatic and chronic stressors, especially during key periods of child development (5, 97, 110, 127).

Taken together, these facts underscore the importance of complementing clinical approaches to mental health with population-based approaches that simultaneously affect entire groups of people. As Eaton (52) notes, the field “must move beyond a narrow focus on clinical interventions to embrace the impact of community and population dynamics in promoting mental health, preventing mental illnesses, and fostering recovery” (p. 511). Although enthusiasm is increasing around the concept of population-based approaches to mental health (126), there is ambiguity about who is best positioned to implement these approaches, what exactly these approaches entail, and which approaches are most supported by evidence. This review seeks to provide clarity in these areas and structure future research and practice in population mental health.

The scope of the review is limited to mental health and does not include substance use disorders or developmental disabilities. Although all three issues are codified in the *Diagnostic and Statistical Manual of Mental Disorders (DSM)* and often considered under the same umbrella of “behavioral health,” the review focuses exclusively on mental health because the approaches best-suited to address each of these issues are distinct. The review is also focused on population-based approaches across three broad domains—public policy, public health practice, and health care system design—and does not focus on strategies for specific settings and populations (e.g., children in schools, people in prisons, older adults in elder care facilities). Reviews of such setting- and population-specific strategies are provided elsewhere (36, 52).

**Behavioral health:**

an umbrella term that refers to both mental health and substance use issues

## DEFINING A POPULATION-BASED APPROACH TO MENTAL HEALTH

Although much has been written about the notion of population mental health (36, 78, 94, 126) and the closely related concept of public mental health (44, 52), a concrete definition of “population-based approaches to mental health” does not exist. Drawing from scholarship about population mental health, as well as that related to “population health” terminology (49, 136), we define population-based approaches to mental health as nonclinical interventions and activities intended to improve mental health outcomes, and the determinants of these outcomes, among a group of individuals that are defined by shared geography, sociodemographic characteristics, or source of clinical services utilization.

The text below describes the rationale for the definition’s three main elements: interventions and activities, outcomes and determinants, and populations.

### Defining Interventions and Activities

Population-based interventions must be nonclinical and thus exclude direct mental health services (e.g., psychotherapies and pharmacological therapies) to individual patients. This criterion is consistent with notions of what a population-based approach to health has historically entailed and the Public Health Accreditation Board’s criteria for activities that are considered population based when accrediting public health departments (115). Health care system-level interventions that address mental health, however, can be considered population-based because the intervention is at the system, as opposed to the clinical, level (136). Social, economic, and environmental policies are also considered population-based interventions because they affect groups of people simultaneously, and psychiatric epidemiology studies indicate that a wide range of public policies affect mental health (detailed below). The definition includes mention of activities to encompass practices that monitor mental health outcomes and determinants without intervening on them.

**Psychiatric epidemiology:**

study of the incidence, prevalence, distribution, causes, and consequences of mental health conditions across people, space, and time

The interventions must also be implemented with the intent of addressing mental health outcomes or their determinants. This requirement stems from dictionary definitions of “approach”—“a way of doing or thinking about something such as a problem or a task” (Oxford; https://www.oxfordlearnersdictionaries.com/us/definition/american_english/approach_2) or “the taking of preliminary steps toward a particular purpose” (Merriam-Webster; https://www.merriam-webster.com/dictionary/approach)—which specify action related to a specific goal. However, this review draws from research about population-based interventions that affect mental health outcomes or determinants without the explicit intent of addressing them. These interventions are relevant because they could be implemented with the explicit intent of improving population mental health. The review also draws from research about population-based interventions that negatively affect mental health. Identifying these deleterious interventions is important because reforming them can produce benefits for population mental health.

### Defining Outcomes and Determinants

Mental health outcomes can be conceptualized in many different ways—ranging from meeting full diagnostic criteria for a disorder in the *DSM*, to symptom severity of a disorder regardless of whether full *DSM* criteria are met, to general emotional distress, and across a continuum spanning from languishing to flourishing (83). Consistent with widely accepted notions of population health (85, 126), determinants of mental health are included in the definition. These include proximal determinants (e.g., poor sleep quality, exposure to traumatic stressors) as well as distal determinants (e.g., built environments with excessive ambient light and noise at night, high rates of community violence), which are the causes of the causes.

### Defining Populations

As Kindig (85) describes in his review of “population health terminology,” a population “refers to a group of individuals, in contrast to the individuals themselves, organized into many different units of analysis” (p. 142). In this review, the focus is on groups that share a geographic region (e.g., state or country), sociodemographic characteristics (e.g., ethnic or sexual minorities), or shared source of clinical service utilization (e.g., hospital, health care system). These types of groups encompass how populations are typically conceptualized in both public health (36) and health care (49).

## HISTORY OF POPULATION-BASED APPROACHES TO MENTAL HEALTH IN THE UNITED STATES

Historical debates about America’s mental health care system and how best to meet population mental health needs probably sound familiar to those engaged in contemporary discussions about improving mental health care. For example, questions about whether populations are best served by the creation of state-run institutions (“mental asylums,” as they were once known) or in community-based settings have been debated among American mental health professionals and policy makers since the late eighteenth century (65).

For most Americans, asylums are viewed as barbaric relics of the past, places where people were locked away under state control, mistreated, and forgotten. These historical experiences are why recent proposals to bring back asylums and institutionalized care have generated significant controversy, even in the wake of the failure of a mental health care system to absorb the more than half-a-million patients who were once housed in state psychiatric facilities (132). As proponents of a return to asylum-based care point out, however, these changes have led to transinstitutionalization: Some patients are now cared for in community-based settings, and still others in hospital emergency departments. Meanwhile, across the United States, prisons and jails have become the default centers of care for patients with mental health needs. Approximately 40% of America’s incarcerated population has been diagnosed with a mental illness (25, 132).

Before the nineteenth century and the rise of an asylum-based care system, mental health concerns were viewed primarily through economic and moral lenses: Could a sick individual and their family economically support themselves in colonial society, and what was the responsibility of the community to support individuals who could not support themselves or be supported by family? Thus, most of the care for people who were identified as having some type of mental health need—at that time they would likely have been labeled with the catch-all term “insane”—took place at home with some community economic support, though jails were still a place to house disruptive individuals (65).

Over the course of the nineteenth century, care for mentally ill individuals, then known as “lunatics” or “distracted persons,” increasingly became a public concern as the social disruption brought about by rapid population growth, urbanization, and a changing economic system increased the visibility of and public attention to mental health issues (65). A concomitant shift in the nation’s *zeitgeist* witnessed Jacksonian-era America (the 1820s to 1840s) move away from viewing mental health concerns as largely private matters to embracing a public response to a wide range of perceived social deviances, which included individuals suffering from “lunacy.” Thus, social reformers of that era set their sights on creating structures to support and redeem the “unfit,” which would include a disparate system of local and state-run asylums that would care for, through much of the twentieth century, a significant number of Americans in need of mental health services (124).

During the nineteenth century, the field of psychiatry emerged to care for asylum patients and to address population mental health concerns. And for most of the nineteenth century, psychiatrists worked largely in these public institutions (73). By the turn of the twentieth century, progressive reformers, along with psychiatrists and other mental health professionals, advocated reforming asylums and launched the mental hygiene movement, dedicated to cultivating healthy lifestyles across the population, preventing mental illness, and providing mental health care and treatment for those in need (95).

The National Committee for Mental Hygiene, formed by social reformers and reform-minded psychiatrists such as Adolf Meyer, considered one of the founders of modern psychiatry, helped shift the national debate about mental health care away from its sole focus on asylum-based treatment toward public mental health and a focus on prevention and etiology. One challenge of this new approach was how it fueled the examination of perceived population-level social pathologies (e.g., homosexuality). Such conditions became medicalized and pathologized, requiring psychiatric treatment and causing various degrees of harm to both the individuals who were mistreated by the profession and populations that faced stigma because of an identification with a specific diagnosis (66, 95). Eugenic ideology and policy were the most extreme expressions of such thinking, and the association between racial hygiene and mental fitness in the opening decades of the twentieth century led to sterilization policies across the United States, helping also to inspire Nazi eugenic practices that systematically murdered the “mentally defective” and disabled (the T-4 program) during the Hitler regime (35).

But not all work in community-based psychiatry sought to pathologize populations. In fact, from the Progressive Era through the post–WWII period, work conducted by psychiatrists, epidemiologists, and sociologists, using both social science and epidemiological methods, examined the social determinants of population mental health and characterized the relationship between mental health conditions and the nature of communities (24, 95). Pioneering work by Faris & Dunham (56) investigated the social determinants of mental health by neighborhood (primarily in Chicago) and the overall impact of urban life on mental health. Community surveys, including the Midtown Manhattan Study in the 1950s and the Health Opinion Survey in Nova Scotia in 1959, examined mental health in different sociocultural settings. These studies and others found that mental illness symptoms were widespread in all populations, though the burden of psychiatric disease did vary between groups and was related to social and economic determinants (24).

**Social determinants of mental health:**

conditions in which people live, learn, work, and play that affect mental health outcomes

This important early work in psychiatric epidemiology provided policy makers with data that helped categorize mental health conditions and quantify population mental health trends, thereby providing foundations for the emerging field on which to build. But the standardization of clinical nosologies for mental health, which would emerge slowly during the second half of the twentieth century and would be systematized in the *DSM-III*, published in 1980, would unfortunately continue to move the field away from an interest in the etiological and sociocultural factors of mental health toward a medical model of psychiatric illness that focused on individuals and the symptomatology of mental illness (3). Pharmacological, rather than structural or community-based, interventions thus became the primary focus of this new biologically rooted psychiatry (73).

The Surgeon General’s Report on Mental Health in 1999, the first surgeon general’s report focused on mental health, drew renewed attention to population mental health challenges by explicitly stating that “mental health is fundamental to health and human functioning” (112, p. 477). Furthermore, the report acknowledged that mental health programs, just like other health programs, are “rooted in a population-based health model” (112, p. 27). In the opening decade of the twenty-first century, novel approaches to measuring the prevalence of mental illness instilled new confidence in psychiatric epidemiology’s ability to observe and address mental health issues at the population level (73).

## POPULATION-BASED APPROACHES TO MENTAL HEALTH AND THEIR EVIDENCE

Table 1 shows three broad domains in which population-based approaches to mental health can be implemented, identifies actors that are key to implementing these approaches, and summarizes core activities to these approaches. The text that follows provides detail about these approaches and evidence of their effectiveness.

### Social, Economic, and Environmental Policy Approaches

Public policies affect populations, and nearly every policy imaginable could plausibly impact mental health. Research about the social determinants of mental health (2, 130, 147) and psychiatric epidemiology studies have elucidated pathways through which policy exposures might affect mental health (82), such as gene × environment interactions (129), psychosocial stress (139), and disrupted sleep (11). In general, much more is known about the negative impacts of policies on mental health than about how policy can improve population mental health. This section provides a high-level summary of goals that can be advanced by public policy to improve the determinants of population mental health. The policy areas and approaches discussed in this section are not exhaustive, and each could be the focus of its own review.

**Gene × environment interaction:**

when a person’s genetic predispositions for an outcome (e.g., mental illness) vary according to the environmental conditions to which they are exposed

### Reduce the incidence of traumatic events.

Exposures to traumatic events are well-established risk factors for post-traumatic stress disorder, depression, and a range of other mental health problems (98). Thus, policies that reduce the incidence of, and exposure to, traumatic events could substantially improve population mental health. Although not adopted with the explicit intent of improving mental health, evidence-based policies that prevent violent crime and accidental injuries—common traumatic events that carry high risk for mental health problems (84, 116)—might have the greatest impacts. For example, an agent-based model simulated the independent and combined effects of policies that reduce violent crime exposure and increase access to mental health services (29). In addition to direct exposure to traumatic events, hearing about traumatic events can increase psychosocial stress and mental health risk, such as demonstrated by research on the effects of police killings on the mental health of black Americans (21). Youth bullying is also a type of potentially traumatic event that has long-term mental health consequences (6). State and local policies that prevent bullying at school and online could produce mental health benefits, but evidence on the effects of these policies is lacking.

**Stress:**

an individual’s mental, emotional, and physical response to demands or changes that require an adjustment

**Potentially traumatic events:**

experiencing, witnessing, or hearing about death, threatened death, actual or threatened serious injury, or actual or threatened sexual violence

### Reduce the incidence of adverse childhood experiences.

Cumulative exposure to adverse childhood experiences (ACEs) increases risk for adult mental health problems (39, 75). For example, a meta-analysis found that adults with ≥4 ACEs had 4.4 times higher odds of depression than adults with 0 ACEs (75), and estimates indicate that approximately 25% of mood disorders among US adults are attributable to the ACEs of childhood sexual abuse, physical abuse, and witnessing domestic violence (1). Thus, similar to traumatic events, policies that reduce exposure to ACEs could improve population mental health. The National Conference of State Legislatures (19) has compiled a list of evidence-supported policies that can be adopted to prevent exposure to ACEs, such as funding nurse–family partnerships, raising the minimum wage, and extending earned income tax credits.

**Adverse childhood experiences:**

direct and indirect exposure to violence, abuse, neglect, and other forms of extreme stress before the age of 18—originally operationalized as ten indicators of “household dysfunction”

### Modify the built environment.

Moderate evidence has shown that public policies can be used to improve population mental health by modifying three features of the built environment: green space, ambient light at night, and ambient noise (55, 143). Exposure to green space, especially in urban areas, is associated with positive mental health, and policies that increase exposure could produce benefits by reducing stress, increasing physical activity, and preventing violent crime exposure (48, 89). For example, a cluster randomized-controlled trial of vacant lot greening found that the intervention significantly reduced feelings of depression, poor mental health, and neighborhood violent crime, with the strongest effects in low-income neighborhoods (23, 133). However, these findings are in contrast to a review of earlier intervention studies that concluded that there was limited evidence to support the mental health benefits of such built environment interventions (103).

Excessive exposure to ambient light at night can increase one’s risk of mental health problems by compromising sleep quality (17, 33, 89, 111). Policies that reduce the amount of short-wavelength light from LED bulbs on street lights, dim street lights during off-peak nighttime hours, encourage the use of shields on street lights to reduce light scatter, and incentivize the instillation of blackout curtains could potentially produce benefits for population mental health (17). However, empirical studies assessing the effects of such policies are lacking. Excessive exposure to ambient noise at night also disrupts sleep (113) and could increase mental health risk, although few well-designed studies have examined these associations (51, 125, 142). Policies that fund the erection of sound barriers and regulate the noise of engines—such as those of heating and cooling systems, trucks, and construction equipment—could minimize exposure to deleterious ambient noise (104).

### Reduce financial and housing insecurity.

Associations between socioeconomic position and mental health are extremely complex, but evidence has shown that policies that promote financial and housing security among low-income populations could produce mental health benefits by reducing exposure to chronic stressors (144). Financial concerns are a significant source of stress for 81% of US adults ages 18–21 and 64% among other adults (4); increasing the minimum wage and extending earned income tax credits are two policies that could reduce stress and improve mental health (22, 72, 91, 114, 123). Analyses of repeated cross-sectional Behavioral Risk Factor Surveillance System (BRFSS) data found that increases in state minimum wages were associated with improved self-rated mental health among women but not among men (72). A $1 increase in minimum wage was independently associated with a 1.9% reduction in state suicide rate, preventing approximately 8,000 suicides between 2006 and 2016 (61). The mental health benefits of improved financial security have also been demonstrated by natural experiments assessing the effects of increases in household income resulting from casinos opening on Native American territories (148), notably the longitudinal investigations from the Great Smoky Mountains Study (42, 43). Outside of the United States, quasi-experimental studies evaluating the mental health impacts of the 1999 National Minimum Wage policy in the United Kingdom found mixed results (87, 123).

Public policy can also reduce stress among low-income groups and improve population mental health by reducing housing insecurity, such as through increasing public housing subsidies (54). For example, quasi-experimental studies have found that public housing subsidies improve mental health among low-income adults (58) and children (59). Home foreclosures have consistently been found to produce consequences for mental health (141), and policies that prevent foreclosures—such as those similar to the federal Making Home Affordable program—could produce mental health benefits.

### Reduce structural stigma toward people with mental illness and members of other social groups.

Structural stigma, which is often codified in policies, negatively affects population mental health though discrimination that inhibits access to resources and by fostering feelings of social exclusion and stress among socially marginalized groups (68). Identifying and reforming policies that create structural stigma is a strategy to improve population mental health. In terms of structural stigma toward people with mental illness, examples of policies to reform include those that prohibit people with mental illness from holding public office or practicing medicine (40, 41). State laws and administrative policies that enhance and promote full compliance with federal mental health parity laws could also reduce structural stigma toward people with mental illness (47).

In terms of other socially marginalized groups, numerous studies have demonstrated associations between policies that affect structural stigma toward sexual minorities and the mental health of these groups (67, 69). For example, a quasi-experimental study of the impacts of same-sex marriage laws found that the laws reduced suicide attempts by sexual minority youth (122). Policies that support structural stigma toward immigrants in the United States have been shown to negatively impact Latino mental health (70). Structural racism, a concept similar to structural stigma, has been shown to have major consequences for the mental health of Blacks in the United States (12).

**Structural stigma:**

societal conditions, cultural norms, and policies that constrain the opportunities, resources, and well-being of socially marginalized groups

**Structural racism:**

laws and system-level policies that limit racial minorities’ access to social and economic opportunities

### Public Health Practice Approaches

The United States has 59 state and territorial public health departments and more than 2,800 local public health departments (LHDs), all of which have a mandate to protect and promote population health. Although the structure and function of public health departments vary across the United States, most operate separately from their jurisdiction’s mental health agency (see the sidebar titled Organizational Structure of Relationships Between Public Health Departments and Mental Health Agencies). Although mental health agencies in some jurisdictions have embraced a population-based approach—New York City and Philadelphia, for example—mental health agencies are usually narrowly focused on providing clinical services to individuals with diagnosable mental illnesses. All public health departments, in contrast, embrace a population-based approach by carrying out the 10 Essential Functions of Public Health (20).

Over the past decade within the field of public health, there has been substantial interest in integrating mental health into public health practice. For example, mental health is the focus of 12 Healthy People 2020 objectives, “mental and emotional well-being” is a priority of the National Prevention Strategy, and the Centers for Disease Control and Prevention’s chronic disease action plan lists “[d]evelop[ing] strategies for integrating mental health and mental illness into public health systems” as an objective (28). A logic model has been developed to guide such integration (90). Despite the enthusiasm about integrating mental health into mainstream public health practice, surprisingly little empirical research has examined how or why mental health is integrated into public health practice or assessed the impact of public health department activities on population mental health outcomes or determinants.

### Scope of public health department activities to address population mental health.

Limited data exist about public health department engagement in mental health. At the state/territorial level, some information comes from the Association of State and Territorial Health Officials’ Profile Survey. The 2016 survey found that only five state/territorial public health departments identified mental health as one of their top five priorities (7). At the local level, more detailed information is available from the National Association of County and City Health Officials’ (NACCHO’s) Profile Study survey. These data have been used to generate national estimates of the prevalence of correlates of LHD engagement in broad categories of population-based mental health activities (118, 119, 128). Consistent with state-level data, the survey indicates that LHDs engage in mental health as an exception, not the norm.

The proportion of LHDs that engage in population-based mental health activities (see the sidebar titled Prevalence of Local Health Department Engagement in Population-Based Mental Health Activities) is substantially lower than the proportion that engage in the same activities to address physical health (36). For example, while 20.3% of LHDs engage in population-based mental illness prevention activities, 60.2% engage in population-based physical chronic disease prevention activities; likewise, 28.2% of LHDs engage in policy/advocacy in the area of mental health, while 55.8% do in the area of physical chronic disease (117). Analyses of 2013 Profile Study data found that 44.2% of LHDs did not perform any activities to address mental health (118).

Qualitative data and case studies offer more detailed information about the specific activities that LHDs perform to promote population mental health (36, 105, 121). These activities include conducting trainings about trauma-informed practice and monitoring population mental health status. A list has been published of evidence-based, population-based mental health interventions that both are recommended by the US Community Preventive Services Task Force and satisfy Public Health Accreditation Board requirements, such as advocating for comprehensive state behavioral health parity laws and promoting access to home-based depression services for older adults (106). It would also be within the scope of public health department practice to implement or advocate for many of the policy approaches detailed above (e.g., vacant lot greening, violence prevention). Furthermore, health departments can implement evidence-based communication campaigns to reduce interpersonal stigma toward people with mental illness (99)—which is highly prevalent among the US general public (16, 107) and policy makers (120)—and cultivate public support for policies that reduce structural stigma.

**Behavioral health parity laws:**

statutory requirements for health insurance companies to provide equal benefits coverage for behavioral health and medical/surgical services

### Barriers and facilitators to public health department engagement in population mental health.

Few studies have examined barriers and facilitators to public health department engagement in mental health. That which is known comes from one qualitative study focused on the topic (121) and analyses of Profile Study data focused on the correlates of mental health activities (118, 119, 128). Many barriers to LHD engagement in mental health stem from LHD officials’ lacking content expertise and training in mental health (121). A 2014 analysis of the curricula of 48 accredited schools of public health found that only 15% offered concentrations in mental health (145). Impediments also relate to the interagency dynamics between LHDs and local mental health agencies, such as fears of infringing upon the “turf” of another agency (121). Limited financing and scarce resources at LHDs are also barriers, especially in jurisdictions where mental health is “someone else’s job” (121, p. 70).

Facilitators to LHD engagement in mental health might be emerging, however, as macrolevel trends in public health practice—including health department accreditation—could push mental health into mainstream public health practice. Bommersbach and colleagues (20) detail how LHD accreditation can serve as an impetus for LHDs to address mental health, especially in light of a 2015 Public Health Accreditation Board policy change, which stated that population-based mental health activities will be considered when reviewing accreditation applications (115). One specific way that accreditation could facilitate LHD engagement in mental health is through the requirement for LHDs to conduct community health assessments as part of the accreditation process. Mental health is often identified as a top community priority through these processes (121), similarly to how it is identified in community health needs assessments conducted by nonprofit hospitals (50).

### Effects of public health department activities on population mental health.

While a growing body of evidence has demonstrated links between the activities of LHDs and physical health outcomes (64, 76, 96), very little evidence exists about the impact of LHD activities on population mental health. That which is known comes from three studies conducted by Chen and colleagues that assessed the impacts of LHD mental illness prevention programs (30–32). Two studies of LHDs in Maryland found that these prevention activities were associated with significantly lower rates of preventable hospitalizations among individuals with anxiety and/or depression and coexisting chronic conditions (30) and 30-day all-cause hospital readmission rates (31). A related national study found that these prevention activities were independently associated with $824 per-person lower health care expenditures in LHD jurisdictions (32). A major limitation of these studies, related to limitations of the Profile Study data set, is the absence of detail about which specific mental illness prevention activities LHDs performed.

### Strategies for health departments to monitor and assess population mental health.

Population mental health surveillance and monitoring are essential to effectively designing and deploying population-based interventions to improve mental health (37), especially during periods of acute stress, such as disasters and economic downturns. While many publicly available data sources can be used to generate population-level estimates of mental health status at state and local levels (8), the BRFSS is the primary data source for many health departments (see the sidebar titled Mental Health Items Used in the Behavioral Risk Factor Surveillance System). The Council of State and Territorial Epidemiologists developed a list of mental health indicators that it recommends health departments monitor using the BRFSS and the National Survey of Drug Use and Health (46). However, some LHD officials have expressed that these data sources are inadequate for their needs because they are often unable to produce precise estimates at the level of LHD jurisdictions (e.g., counties or cities) or among subpopulations within these jurisdictions (121).

The use of big data has also emerged as a potential strategy for health departments to monitor population mental health. Health department syndromic disease surveillance systems—which use chief complaint emergency department data, traditionally with the primary goal of identifying disease outbreaks—can be an efficient way to monitor population mental health. Algorithms have been developed to identify mental health problems with high sensitivity and specificity (63, 134). Google Trends data also demonstrate promise to monitor trends in population mental health (109), including how mental distress varies between seasons (10) and during periods of heightened stress (9). Natural-language processing techniques are also being developed to monitor population mental health using social media data (38), although the ethical considerations to using these data are complex.

In addition to monitoring population mental health, health departments can assess the potential impacts of proposed policies and planning decisions by considering mental health in health impact assessments (HIAs). A systematic review of HIAs conducted in the United States between 1993 and 2013 found that 73.1% assessed mental health impacts, and 64.0% predicted effects that a proposal might have on population mental health (93).

### Health Care System Approaches

Health care delivery systems are increasingly supplementing their clinical mental health service activities with population-based strategies to improve the mental health of those they serve. This shift reflects changes in health care financing that incentivize population health investments, many of which have been invigorated by the Affordable Care Act (15, 101). Many of these systems-level strategies are novel, and evidence of their effects is still emerging.

### Enhance the effectiveness of clinical mental health services.

Health care systems often implement systems-level strategies to improve the effectiveness of clinical mental health services. These interventions may be quality improvement initiatives such as training and coaching to promote the implementation of evidence-based practices (92), supports for measurement-based care to help providers evaluate patient progress and update treatment plans accordingly (60), and audit-feedback systems based on administrative data, such as feedback to reduce unnecessary antipsychotic prescribing (138). Health care systems can also support providers and improve patient outcomes by implementing care coordination programs. These can include the use of care managers who help ensure that patients can access needed mental health care (135) and analytic efforts that use electronic health record data to help health care systems identify patients who might need mental health services (131).

Other strategies focus on integrating mental health services into primary care. The collaborative care model is an effective and well-studied example of this approach (140). Other effective models include the primary care behaviorist model, which integrates a psychologist into primary care (57), and the healthy steps for young children model, which integrates a developmental specialist into pediatric primary care (102). Some health care systems also work to engage individuals in community-based settings to provide early intervention services, promote sustained engagement with care, and initiate intensive services for complex needs. Community Partners in Care is an example of an effective strategy for health care systems to partner with trusted community leaders to engage underserved populations (146).

Mental health can also be integrated into the practices of learning health care systems, which dynamically improve through rapid cycles of innovation, evaluation, and dissemination (88). For example, a continuous learning system is being developed to improve outcomes for patients with psychotic disorders through the Early Psychosis Intervention Network (71).

### Provide consultation and training to community partners.

Health care systems can also deploy mental health providers to provide consultation and training to community-based partners and extend the impact of clinical interventions. For example, the Early Childhood Mental Health Consultation Intervention involves a mental health provider partnering with a preschool teacher to help address the mental health needs of specific students through regular check-ins (62). Health care system–affiliated mental health providers can also help schools implement classroom-wide evidence-based interventions, such as the Good Behavior Game (81). Similarly, health care system–affiliated providers can assist with the development and implementation of evidence-based workplace mental health interventions in the communities they serve (80).

### Employ paraprofessionals to address social needs and promote recovery.

Health care systems can provide nonclinical services that are effective at addressing prevention and recovery in mental health. Peer support specialists—people who have successfully lived with mental illness and help others recover in a formal and compensated capacity—can serve various functions within health care delivery systems, such as assisting with care navigation (34). Health care systems can also support prevention and recovery in mental health by employing other types of paraprofessionals, such as community health workers who can deliver nonclinical mental health interventions [e.g., psychoeducation or stress management training (14)]. Paraprofessionals can also help people with mental illness overcome barriers to recovery, such as through evidence-based, supported employment interventions that use a combination of clinical and nonclinical staff to help individuals access and maintain employment (86).

**Recovery in mental health:**

a process through which people with mental illness gain hope, engage in an active life, and achieve personal autonomy and social identity

### Participate in accountable communities for health initiatives.

In the accountable communities for health model, a third-party nonprofit coordinates efforts among stakeholders in a community to advance a common goal, which can be population mental health (45). Health care systems can play a prominent role in these endeavors. With the accountable communities for health model, health care systems can invest to build capacity for collaboration around mental health and even invest in the other organizations to support complementary interventions. Some nonprofit health care systems make these investments to demonstrate community benefit, and some health care systems have created investment funds to enhance the capacity of community-based organizations to address population mental health (27).

### Partner with public health departments.

Health care systems can also partner with public health departments and share data to inform planning activities. For example, to satisfy their community-benefit requirement, nonprofit hospitals can partner with health departments to fund population-based mental health interventions in the communities they serve. A 2015 analysis found that 71% of nonprofit hospitals identified mental health as a priority in their community health needs assessments, and 49% identified mental health activities in their implementation plans (50). Health care systems can also share deidentified data to help public health departments and policy makers monitor and respond to emerging mental health issues (63, 131, 134). For example, a Baltimore hospital used routinely collected data about maternal well-being to identify mental health impacts of the 2015 riots (149). Like public health departments, health care systems can also advocate for evidence-supported public policy changes that have the potential to improve population mental health.

## FUTURE DIRECTIONS

Near-term future directions include addressing emergent risks to population mental health and capitalizing on new opportunities to improve it. In terms of risks, there is an urgent need to prevent and mitigate the mental health effects of climate change (53). There is also a need to better understand, and intervene on, the mental health consequences of harmful social media exposures and stressful online interactions (e.g., cyberbullying), especially among youth and adolescents (6). However, there are also opportunities to use technology to improve population mental health. Smartphones can plausibly identify people experiencing serious mental distress, connect them to evidence-based mobile interventions, and help facilitate care management (77).

Widespread implementation of population-based approaches to mental health is likely to require a collective shift in thinking at the societal level—from a view that conceptualizes mental health as an individual issue that is exclusively within the purview of psychologists and psychiatrists to one that conceptualizes mental health as a public health issue that actors and organizations across all sectors have a responsibility to address. Structural changes related to financing, training, and accreditation are also likely needed to institutionalize population-based approaches to mental health across sectors (18, 108). More research is needed to better understand the impacts of population-based approaches to mental health to ensure they are effective and reduce, not exacerbate, disparities in mental health problems between socially disadvantaged and advantaged groups. While more evidence is needed, the state of the science is sufficient to recommend specific courses of action to improve population mental health.

## Figures and Tables

**Table 1:** Population-based approaches to mental health: domains, actors, and activities

Domain	Social, economic, and environmental policy interventions	Public health practice interventions	Health care system interventions
Key actors	Elected and administrative policy makers	Public health department officials	Health care system, hospital leaders
Core activities of approach	Reduce exposure to traumatic stressors and adverse childhood experiences Modify the built environment to increase access to green space and reduce exposure to ambient light and noise pollution at night Reduce financial and housing insecurity Reduce structural stigma toward people with mental illness and other socially marginalized groups	Advocate for public policies that would improve population mental health Implement communication campaigns to reduce mental illness stigma Conduct outreach to increase access to community-based mental health services Convene stakeholders and coordinate cross-sector mental health initiatives Monitor population mental health and prospectively assess the potential mental health impacts of policy and planning proposals	Enhance the effectiveness of clinical mental health care services Provide mental health consultation and training to community partners Employ paraprofessionals to address the social needs of people with mental illness and support recovery Participate in accountable communities for health initiatives Share deidentified data to inform public health practice, policy, and planning decisions

## SUMMARY POINTS

1. The provision of clinical mental health services, in isolation, is not a sufficient strategy to maximize population mental health.
2. Population-based approaches to mental health can be defined as nonclinical interventions and activities intended to improve mental health outcomes, and the determinants of these outcomes, among a group of individuals who are defined by shared geography, sociodemographic characteristics, or source clinical service utilization.
3. Public policies that are not implemented with the explicit intent of addressing mental health, but reduce the incidence of traumatic events and limit exposure to psychosocial stressors, might have the greatest impacts on population mental health.
4. The highest-quality evidence about the impacts of population-based approaches to mental health comes from natural experiments of public policies and cluster randomized-controlled trials of built environment interventions.
5. Reforming policies that produce structural stigma toward people with mental illness and other socially marginalized groups is critical to creating a sociopolitical context that promotes population mental health.
6. There is a strong rationale for public health departments to implement population-based approaches to mental health, but public health department involvement in this area is limited and few studies have assessed the impacts of health department activities on population mental health.
7. Changes in health care financing create incentives for health care systems to make investments in population mental health, but such investments have been fairly limited and evidence of their effectiveness is generally lacking.

## ORGANIZATIONAL STRUCTURE OF RELATIONSHIPS BETWEEN PUBLIC HEALTH DEPARTMENTS AND MENTAL HEALTH AGENCIES

Sixty-three percent of state public health departments in the United States are located in the same umbrella organization as the state mental health agency, and 19% of LHDs are part of a larger health and human services agency, which is likely to also house the local mental health agency.

## PREVALENCE OF LOCAL HEALTH DEPARTMENT ENGAGEMENT IN POPULATION-BASED MENTAL HEALTH ACTIVITIES

The 2016 NACCHO Profile Study estimates the prevalence of select population-based mental health activities performed by LHDs:^1^

- “Social and mental health” data or information included in the most recent LHD community health assessment = 84.9%
- LHD actively involved in policy/advocacy activities in the area of “mental health” in the past two years = 28.2%
- Population-based primary prevention activities performed to address “mental illness” performed by LHDs in the past year = 20.3%
- New local public health ordinance/regulation was adopted or substantively revised related to “mental health” in the past two years = 0.7%
- Assessed the gaps in access to “behavioral health” services = 50.4%
- Implemented strategies to increase accessibility of existing “behavioral health” services (e.g., referrals) = 37.9%
- Implemented strategies to target “behavioral health” service needs of underserved populations = 30.4%
- Evaluated strategies to target “behavioral health” service needs of underserved populations = 29.3%
- Addressed gaps in access through direct provision of “behavioral health” service = 17.4%

^1^Percentages are weighted using Module 2 weights to produce nationally representative estimates. “Behavioral health” services are defined in the survey as “psychological, substance abuse, mental health” services.

## MENTAL HEALTH ITEMS USED IN THE BEHAVIORAL RISK FACTOR SURVEILLANCE SYSTEM

The core section of the 2018 BRFSS questionnaire includes six mental health items that span four domains.

- Current frequent mental distress:
  - “Now thinking about your mental health, which includes stress, depression, and problems with emotions, for how many days during the past 30 days was your mental health not good?” (Module 2, continuous)
- Lifetime history of depression:
  - “Has a doctor, nurse, or other health professional ever told you that you had any of the following? … a depressive disorder (including depression, major depression, dysthymia, or minor depression)?” (Module 6, dichotomous)
- Current depressive symptoms:
  - Over the last 2 weeks, how often have you been bothered by having little interest or pleasure in doing things? (Module 9, ordinal)
  - “Over the last 2 weeks, how often have you been bothered by feeling down, depressed or hopeless?” (Module 9, ordinal)
- Current anxiety symptoms:
  - “Over the last 2 weeks, how often have you been bothered by feeling nervous, anxious or on edge?” (Module 9, ordinal)
  - “Over the last 2 weeks, how often have you been bothered by not being able to stop or control worrying?” (Module 9, ordinal)
